# Research protocol of the Laval-ROSA Transilab: a living lab on transitions for people living with dementia

**DOI:** 10.1186/s12913-023-10248-6

**Published:** 2023-11-14

**Authors:** Isabelle Dufour, Geneviève Arsenault-Lapierre, Maxime Guillette, Nathalie Dame, Marie-Eve Poitras, Marie-Thérèse Lussier, Annie Fortier, Julie Brunet, Julie Martin, Micheline Laverdure, Ginette Brousseau, Howard Bergman, Yves Couturier, Amélie Quesnel-Vallée, Isabelle Vedel

**Affiliations:** 1 School of Nursing, Faculty of Medicine and Health Sciences, Université de Sherbrooke, 3001, 12E Avenue Nord, Sherbrooke, QC J1H 5N4 Canada; 2 Lady Davis Institute for Medical Research, Jewish General Hospital, Montreal, Canada; 3 School of Social Work, Faculty of Letters and Humanities, Université de Sherbrooke, Sherbrooke, Canada; 4 Department of Family Medicine and Emergency Medicine, Faculty of Medicine and Health Sciences, Université de Sherbrooke, Sherbrooke, Canada; 5 Department of Family and Emergency Medicine, Faculty of Medicine, Université de Montréal, Montreal, Canada; 6 Integrated Health and Social Services Centre of Laval, Laval, Canada; 7 Department of Family Medicine, Faculty of Medicine and Health Sciences, McGill University, Montreal, Canada; 8 Department of Sociology, Faculty of Arts, McGill University, Montreal, Canada

**Keywords:** Dementia care, Primary care, Care transitions, Living lab, Learning health system

## Abstract

**Background:**

The Laval-ROSA Transilab is a living lab that aims to support the Laval Integrated Health and Social Services Centres (Quebec, Canada) in consolidating the Quebec Alzheimer Plan. It aims to improve care transitions between different settings (Family Medicine Groups, home care, and community services) and as such improve the care of people living with dementia and their care partners. Four transition-oriented innovations are targeted. Two are already underway and will be co-evaluated: A) training of primary care professionals on dementia and interprofessional collaboration; B) early referral process to community services. Two will be co-developed and co-evaluated: C) developing a structured communication strategy around the dementia diagnosis disclosure; D) designation of a care navigator from the time of dementia diagnosis. The objectives are to: 1) co-develop a dashboard for monitoring transitions; 2) co-develop and 3) co-evaluate the four targeted innovations on transitions. In addition, we will 4) co-evaluate the impact and implementation process of the entire Laval-ROSA Transilab transformation, 5) support its sustainability, and 6) transfer it to other health organizations.

**Methods:**

Multi-methods living lab approach based on the principles of a learning health system. Living labs are open innovation systems that integrate research co-creation and knowledge exchange in real-life settings. Learning health systems centers care improvement on developing the organization's capacity to learn from their practices. We will conduct two learning cycles (data to knowledge, knowledge to practice, and practice to data) and involve various partners. We will use multiple data sources, including health administrative databases, electronic health records data, surveys, semi-structured interviews, focus groups, and observations.

**Discussion:**

Through its structuring actions, the Laval-ROSA Transilab will benefit people living with dementia, their care partners, and healthcare professionals. Its strategies will support sustainability and will thus allow for improvements throughout the care continuum so that people can receive the right services, at the right time, in the right place, and from the right staff.

## Background

Dementia affects around 570,000 people in Canada (150,000 of whom live in the province of Quebec), which is expected to double over the next 30 years [1, 2]. Dementia is defined as progressive, irreversible, and chronic impairments in memory and cognitive function beyond what would be expected in normal aging [3]. The condition is associated with significant disability and dependency and thus impacts individuals, their families, and societies [4, 5].

Dementia is recognized as a public health priority, and various national policies have been adopted to improve equitable access to healthcare and quality of life for people living with the condition and their care partners [6].

In Canada, many provinces have developed, and some have implemented, Alzheimer plans. Many integrate these individuals' care in primary care settings [7]. In particular, the Quebec Alzheimer Plan (QAP) aims to integrate prevention, detection, diagnosis, treatment, and care of people living with dementia (PLWD) into family medicine groups (FMGs), along with other key services (e.g., home care and community services) [8, 9]. The QAP is composed of three implementation phases: 1) local implementation of pilot innovative projects and evaluation (2013–2016); 2) generalization of changes in all FMGs (2016–2020); and 3) consolidation and generalization of interventions on transitions of care (2020 to this day) [8, 10].

Care transitions are “the movements patients make between health care practitioners and setting as their condition and care needs change during the course of a chronic or acute illness” [11]. PLWD are exposed to numerous care transitions as the type and level of services required to meet their needs vary with disease progression, and are complicated by multimorbidity and polypharmacy [4, 12]. Care transitions for PLWD are still often fragmented and lack coordination, resulting in various adverse outcomes, such as increased hospital readmission rates, adverse medical events, and decreased quality of life [13–15].

### Starting point

Since the second phase of the QAP, the 24 Integrated Health and Social Services Centres (CISSS for Centre intégré de Santé et de Services sociaux) of Quebec, Canada are responsible for ensuring its implementation on their territory. The Laval CISSS, involved in the QAP since its first pilot phase, established that their priorities were linked to improving the transitions between FMGs, home care, and community services. These challenges and needs called for an original and rigorous strategy to develop, evaluate and adjust sustainable innovations [8]. It is in this context that the Laval CISSS contacted the Research on Organization of healthcare Services for Alzheimer’s disease (ROSA) team [16], involved in the evaluation of the QAP’s implementation and impact since the beginning. They put together the Laval-ROSA Transilab, a living lab that aims to support the Laval CISSS in consolidating the QAP implementation by improving care transitions and, ultimately, improving the care of PLWD.

**Fig. 1:**
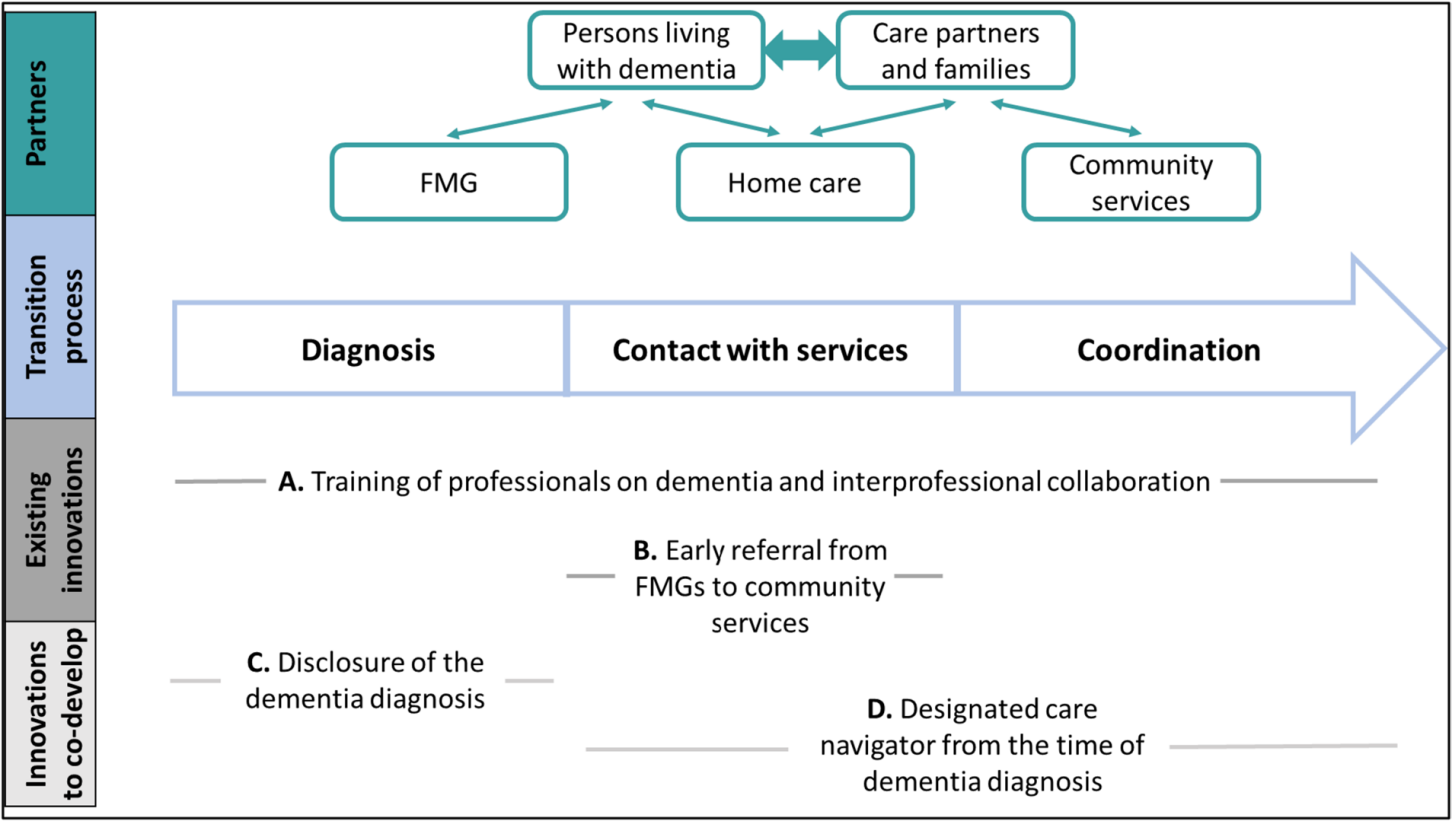
Contextualization of the innovations within the transition process. Abbreviation: FMG, Family medicine group

In 2021, during the discussions between the Laval CISSS, patients’ representatives, and the ROSA team, four transition-oriented innovations were targeted. Two of them are already underway and will be co-evaluated: A) training of professionals in FMGs and home care on dementia and interprofessional collaboration; B) early referral process of PLWD from FMGs to community services. The last two will be co-developed and co-evaluated: C) a structured communication strategy around the disclosure of the dementia diagnosis to PLWD and their care partners; D) a designated navigator model from the time of diagnosis to improve coordination between FMGs, home care, and community services. See Fig. 1 for a contextualization of the innovations within the transition process.

To support these changes, the Laval-ROSA Transilab has six specific objectives. The first three are directly related to the four targeted innovations: 1) co-develop a dashboard for monitoring transitions; 2) co-develop targeted interventions on transitions; 3) co-evaluate the effects and implementation conditions of these innovations in a continuous learning cycle. In addition, we aim to: 4) co-evaluate the impact and implementation process of the entire Laval-ROSA Transilab transformation; 5) support its sustainability; and 6) transfer it to other regions. This paper aims to present the protocol of the Laval-ROSA Transilab and an overview of its processes.

## Methods

### A living lab approach embedded in a learning health system

The Laval-ROSA Transilab is based on living lab, and learning health system (LHS) approaches. A living lab is a “user-centric innovation environment built on every-day practice and research, with an approach that facilitates user influence in open and distributed innovation processes engaging all relevant partners in real-life contexts, aiming to create sustainable values” [17]. Its key elements include: 1) the use of multi-method approaches; 2) engagement of end-users, 3) participation of multiple partners; 4) activities carried out in real-life settings, and 5) co-created environment for innovations [18, 19].

**Fig. 2:**
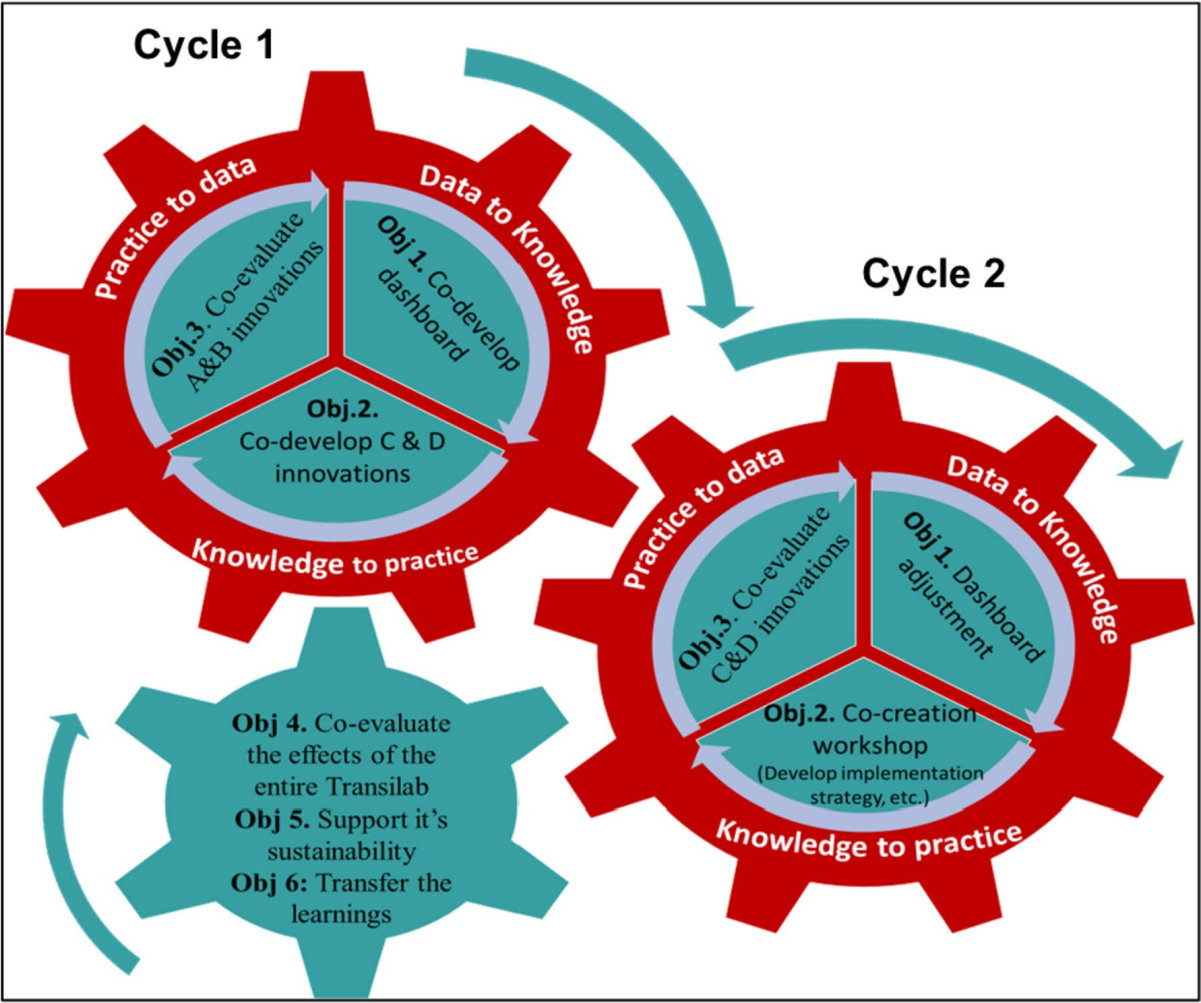
Process of the Laval-ROSA Transilab

LHSs are defined as systems where “science, informatics, incentives, and culture are aligned for continuous improvement and innovation, with best practices seamlessly embedded in the delivery process and new knowledge captured as an integral by-product of the delivery experience” [20]. An LHS approach centers care improvement on developing the organizations’ capacity to learn from their practices [21]. Each LHS cycle has three phases: 1) Data to knowledge (conversion of data from various sources to knowledge that can drive decision-making, improvement, and innovation); 2) Knowledge to practice (the knowledge is applied to support practice innovation and improvement); 3) practice to data (generation of practice-based data) [18]. The Laval-ROSA Transilab comprises two LHS cycles; Fig. 2 shows these cycles and the integration of each objective.

### Objective 1: co-develop a dashboard for monitoring transitions

Within the Transilab, a dashboard for monitoring transitions will be used to visually display information on pre-defined indicators, create knowledge, and influence the practices on the Laval territory. First, we will prioritize indicators of transition for PLWD, according to the 34 indicators of the Framework For Primary Care Quality Indicators For Patients with Dementia that can be operationalized in administrative databases or electronic health records [22]. We will thus run a modified DELPHI technic (two rounds of online questionnaires) with 100 participants, including managers, professionals, patients’ representatives, and community services representatives [23]. Following these results, we will co-design a dashboard for monitoring transitions of PLWD, based on previous work by the ROSA team. We will update the dashboard annually, using two data sources.

The first is health administrative data from the Quebec Integrated Chronic Disease Surveillance System (QICDSS) [24], a provincial database notably providing access to the entire population diagnosed with dementia in the Laval CISSS territory (expected cohort size of 6,000 individuals). The second data source is from the Canadian Primary Care Sentinel Surveillance Network (CPCSSN), an electronic medical record surveillance system that collects various data from FMGs (e.g., diagnosis, medications, labs) [25]. In Quebec, the CPCSSN provides access to approximately 250 PLWD in the Laval CISSS territory. Descriptive analyses (e.g., proportions and frequencies) will be presented along with Sankey diagrams, allowing visualization of care transitions across multiple settings. Ultimately, the Laval CISSS will be better equipped to understand the care trajectories of PLWD within their territory and make more informed decisions regarding care transitions.

### Objective 2: co-develop targeted innovations

For this objective, we will use a participatory methodology [26] for co-designing innovations [27]. The steering committee and QAP representatives will hold a one-day co-design workshop for each LHS cycle. The objective will be to review the dashboard data, discuss the innovations that have been implemented, refine them based on their evaluation and plan the co-development and evaluation of new innovations. These yearly workshops will be essential to fully engage the actors in the co-design and piloting of change. Table 1 present a description of the four innovations (existing and to co-developed).

**Table 1:** Description of the Laval-ROSA Transilab innovations

**Existing innovations**	
A. Training of professionals	
Objective	Improve the knowledge of FMGs and home care professionals about neurocognitive disorders, interprofessional collaboration and services coordination
Importance	Professional development on interprofessional collaboration and services coordination is an area of priority for the Laval CISSS. It comes as a strategy to further develop an integrated and cohesive answer to the needs of PLWD and their care givers, and thus improve the transition process [28, 29]
Description	Three training programs have been developed and implemented **1. FMGs nursing training program**: focuses on dementia care, the role of the nurse, and collaborative work within and outside primary care clinics. In addition to the training, mentoring is offered to promote the integration of the content into practice **2. FMGs social workers training program:** includes the presentation of the QAP, theoretical notions on dementia, the role of social workers concerning dementia in FMGs, and collaborative work within and outside primary care clinics **3. Interprofessional Collaborative Education Unit**: aims at training new professionals in FMGs and home care services in interprofessional collaborations
B. Early referral process from FMGs to community services	
Objective	Formalize the partnership between FMGs and community services, using the Aidance-Quebec referral program
Importance	Persistent challenges were reported regarding service coordination between FMGs professionals and community services. Formalizing the partnership between those services ensure timely access to the needed community services, for PLWD and their care partners [30]
Description	The Aidance-Québec referral program was developed in the province of Quebec [30]. It consists of an online referral tool allowing professionals to refer patients to community services. The PLWD or their care partners are contacted within ten days. The program is already implemented in the CISSS of Laval
**Innovations to be co-developed**	
C. Communication strategy around the dementia diagnosis disclosure	
Objectives	Improve the interview experience during which the diagnosis disclosure for PLWD, their care partners, and FMGs professionals; and ensure the cohesion of the message to improve the understanding of PLWD and their care partners
Importance	The disclosure of a dementia diagnosis involves a lot of discomfort and anxiety for patients, their loved ones, and professionals who often feel inadequately prepared to deal with such a delicate situation. Timely disclosure is a strategy that can facilitate access to treatment and support and decrease emotional burden on PLWD and their families [31, 32].
D. Designated navigator model from the time of diagnosis	
Objective	Establish a simple navigator model to support PLWD and their care partners, and improve coordination between FMGs, home care, and community services The development will be based on the lessons learned during innovations A, B and C, and is still to be established
Importance	Lack of coordination and suboptimal transition processes are still reported in the care of PLWD, leading to negative outcomes (e.g., evitable hospitalisation and institutionalisation). Patient navigation is one way to support PLWD and their care partners throughout the care trajectory, by supporting healthcare system navigation and ensuring better coordination with other health professionals and services (including home care and community services) [33]

*Abbreviations*: *CISSS* Regional Health Boards (CISSS for Centre de Santé et Services sociaux), *FMGs* Family medicine groups, *PLWD* people living with dementia

We already established the co-development plan of Innovation C (disclosure of the dementia diagnosis). Thus, it will be co-created with professionals based in two FMGs of the Laval CISSS. We will organize two co-design meetings to bring out the needs of professionals around dementia diagnosis disclosure, and to develop and validate the intervention, which will consist of a training workshop, based on an experiential approach to learning (e.g., role-playing, feedback). In addition, coaching sessions will be offered to promote integration into their practice. We will build on communication approaches developed to mitigate the negative impact of bad news (i.e., dementia diagnosis disclosure): Calgary-Cambridge approach for practitioner-patient communication [34].

Innovation D (care navigator model) will be co-developed during the second HLS cycle of the Transilab, based on the lessons learned from previous innovations.

### Objective 3: co-evaluate the implementation and impact of the innovations

Following the principles of a living lab approach, the active and continuous participation of our partners is paramount for creating innovation. Thus, they are actively engaged in the research design and evaluation planning. The following sections share the elements of our working plan that are already established.

#### Evaluation of ongoing innovation: training of professionals

To evaluate the impacts and implantation process of the training programs, quantitative survey data will be collected using pre- and post-training assessment tools for professionals attending the training activities. The questionnaires will be constructed using the Kirkpatrick model [35], used to evaluate the results of training and learning programs, and the validated Continuing professional development (CPD)- Reaction tool [36], adapted for the three specific trainings. Retrospective qualitative data collection will be conducted with the trained professionals through individual semi-structured interviews (30 to 45 min) to measure their perceived level of competence and knowledge acquisition and identify enhancement points that can be addressed for future training. In addition, we will conduct approximately 35 post-training observations of clinical appointments via video or audio recording. We will analyze quantitative data from the questionnaires to determine the perceived effects of the training on intention, confidence, and commitment. Matched and unmatched sample analyses will compare pre- and post-training data using the Wilcoxon-Mann–Whitney test and Wilcoxon signed ranks test. An inductive and deductive thematic analysis will be performed on the interview and field observations. Qualitative data will be analyzed in three concurrent streams: condensation, presentation, and verification of conclusions [37, 38].

#### Evaluation of ongoing innovation: early referral process

The early referral process of PLWD to community services is achieved with the Aidance-Quebec referral program implemented in the Laval CISSS [30]. This is a mixed explanatory sequential design [39], using the Consolidated Framework For Implementation Research (CFIR) to guide data collection, as it allows questions to be formulated according to the constructs to be explored [40]. Data on the implementation process will be gathered, including facilitating factors and barriers, and the perceived effect on the intersectoral collaboration between FMGs and community services.

For the quantitative part, users of the referral program from the FMGs and workers of community services on the Laval territory (Alzheimer Society of Laval) will be invited to complete an online questionnaire (n = 85 users), aligned on the CFIR domains (e.g., intervention characteristics, barriers, facilitators). The quantitative data will be subject to descriptive statistical analysis (score, mode, and proportion). Qualitative data will be collected through individual semi-structured interviews with around 20 users of the referral program (30 to 45 min) to identify the perceptions and experiences of the participants and thus add to the understanding of the implementation process. We will submit the qualitative data to content analysis based on transcripts and coded verbatim [41].

#### Evaluation of innovations to be co-developed: dementia diagnosis disclosure

We will co-evaluate the implementation process and the impacts of the intervention in four FMGs, including the two that participated in the co-design process. All practitioners, residents and specialized nurses' practitioners will be invited to take part in the workshops, each gathering 8 to 10 participants. First, they will be invited to complete a pre-workshop questionnaire to evaluate their knowledge of the disclosure process. Post-workshop questionnaires will evaluate the relevance and usefulness of the workshop, the knowledge and skills acquired, and the intention to apply them. The pre- and post-workshop questionnaires will be adapted from the CPD- Reaction tool [36] and use descriptive analyses (pre- and post-workshop scores comparison using the Wilcoxon signed ranks test).

Following the workshops, participants will be offered coaching to facilitate applying the knowledge and communication skills acquired in their clinical practice. The modalities of this coaching will be co-developed with the workshop participants (e.g., case discussions). Participants will be invited to a focus group at the mid and end point of the project to share their impressions of the coaching’s impact on their practice and propose adjustments (2 focus groups by FMGs). Focus group content will be analyzed using a thematic analysis approach [37, 38].

#### Evaluation of innovations to be co-developed: dementia diagnosis disclosure

Using a mixed-methods approach, we will aim to understand the factors influencing implementation and sustainability and to co-evaluate the impact of the innovation. Our processes will be embedded in the CFIR [40]. We plan on using complementary data collection strategies: 1) literature review of internal documents describing the innovation; 2) quantitative questionnaires documenting, amongst other things, the perceived usefulness of the innovation; 3) semi-structured interviews (45–60 min) with three categories of actors (managers of the innovation, users requiring navigation services, and navigators); 4) non-participatory direct observations of one week of navigators' work will also be conducted. Data collection will be repeated once a year (years 2 and 3) to monitor progress.

### Objective 4: co-evaluate the impact and implementation of the entire Laval-ROSA Transilab transformation

The effect of the Transilab will go beyond the four innovations described before. Indeed, we can hypothesize that the governance (see below), the engagement strategy of all partners, and the regular meetings (between professionals, managers, patients’ representatives, and researchers) will have a profound impact on the practices not only of managers and care professionals but also on researchers. Both the impacts and the implementation of the whole Laval-ROSA Transilab will be examined using the conceptual framework of the Evaluation of Complex Interventions [42, 43].

#### Evaluation of the impacts of the Transilab

Two types of quantitative study designs will be conducted to evaluate the overall impact of the Transilab on the clinicians’ attitudes, knowledge, and practice about dementia and on the care transitions of PLWD.

(A) The first study is a repeated measures cross-sectional survey of the FMGs clinicians' knowledge, attitudes, and practices toward dementia and clinicians' satisfaction with home care and community services. Two validated questionnaires will be distributed to: family physicians and nurse practitioners [30]; nurses and other health care professionals [39] of all Laval FMGs, at three measurement times (beginning, middle, and end of the project). These questionnaires assess clinicians' attitudes, knowledge, and professional practices related to the care of PLWD. Means and standard deviations will be calculated (for each dimension and global score) at the three measurement times. Repeated analyses of variance will be done to measure changes throughout the Transilab.
(B) The second quantitative study will be an interrupted time series design with a control group, using the health administrative data from the surveillance system in Quebec (QICDSS) [24]. Our target population will be the community-dwelling persons aged 65 years and over on the Laval territory, with a prevalent diagnosis of dementia before the implementation of the Transilab in January 2022 (anticipated cohort size of 6,000 individuals). The control group will be community-dwelling persons aged 65 years and over from outside the Laval territory, matched on age, gender, type of dementia diagnosis, comorbidity, and the Material and Social Deprivation Index [44]. Using multiple monthly data points, we will compare key transition indicators (among those prioritized in Objective 1) during the pre- (36 months) and post (36 months) implementation of Transilab, using descriptive methods and regression models (e.g., Segmented Regression, Autoregressive Integrated Moving Average).

#### Evaluation of the implementation of the Transilab

A holistic case study with a longitudinal design will be conducted to co-evaluate the implementation and perceived effects of the Transilab, and to identify conditions for its sustainability. Three data sources will be collected and analyzed: 1) we will conduct a documentary analysis of the material used to develop and implement the project (e.g., planning documents, organizational policies, job descriptions, and annual reports). The results will describe the processes of innovations co-creation and how they were adapted to meet the needs of our partners. 2) Semi-directed interviews will be conducted each year with the different groups involved in the Transilab (e.g., members of the steering committee, managers, healthcare professionals, PLWD, and their care partners). The interview guides will be developed using the attributes of innovation and the concepts of Lennox's model on the conditions for scaling up [40]. We will conduct the analysis using the theoretical framework of co-creation of innovation in health care, including analysis of barriers, facilitators, and keys to scaling up [45]. 3) We will conduct participant observations of the Transilab co-creation workshops, including during the co-design of the dashboard (objective 1) and the workshops conducted at each LHS cycle (objective 2), and the co-development of innovations. The observations will focus on the overall design and implementation strategies, adaptation, consultations between the various partners, and the point of view of PLWD and their care partners. A research assistant will extract the relevant information using an observation grid (aiming to extract discussions on overall co-creation and implementation strategies), whose themes will guide the thematic analysis of the data.

### Objective 5: support the sustainability of Laval-ROSA Transilab

The steering committee will work closely with the partners of the Laval CISSS, who have the resources to promote the sustainability of our innovations beyond the planned funding. The Laval CISSS will hire a research agent to facilitate coordination and promote internal sustainability. In addition, we intend to create a community of practice that will bring together various partners with the potential to contribute to the sustainability of the Transilab. Although no data collection will be conducted for this objective, it is crucial.

### Objective 6: transfer these learnings to other regions of the province of Quebec

We will share the knowledge gained from implementing our innovations with three health organizations from various regions of the province of Quebec. In years two and three of the project, we will forward a summary of the results of the Transilab and remain available for discussions if needed. In year three, each partner will be invited to a deliberative workshop to explore the potential of transferability of the Transilab, using the Consolidated Framework For Scale Up [46]. This objective is paramount to support the learning transfer from the Transilab to other health organizations.

### Governance

The Transilab is guided by a participatory methodology [26] and co-management of research [47] approaches. Our steering committee comprises ten people (managers, clinicians, community services representatives, care partners of PLWD from the Laval CISSS, and researchers). The committee will meet quarterly to make strategic decisions regarding the development of innovations and evaluation/research priorities, monitor the progression, and develop the plan to ensure sustainability.

### Ethical approval

This study is approved by the Research Ethics Committee of the Laval CISSS (2023–938). The project received major external funding from the Fonds de recherche du Québec – Santé (2022–2025; 750,000$).

### Timeline

This ongoing study will take place over three years (2022–2025). The protocol was submitted for funding in January 2022 (start of the Transilab Laval-ROSA). Two one-year LHS cycles will take place, with co-development and co-evaluation of innovations within each LHS cycle. The recruitment is expected to be completed in the latter part of 2024.The last few months of 2025 will be dedicated to finalizing the global co-evaluation of the Transilab, its sustainability, and dissemination efforts and mark its end.

### Dissemination

In addition to the dissemination activities planned in objectives 5 and 6 with the Laval CISSS partners and other CISSS, the research results of this research will be disseminated through various local and wide-reaching dissemination strategies, including: 1) diffusion of personalized tools (infographics) on various platforms (e.g., Twitter, ResearchGate, LinkedIn, webpages) and to our partners; 2) media diffusion during key moments (e.g., World Alzheimer’s Day); 3) presentations at national and international conferences, seminars/webinars, and workshops; and 4) publication of at least eight scientific articles in peer-reviewed journals.

## Discussion

The Laval-ROSA Transilab is a living lab aiming to consolidate the achievements of the QAP and extend its development towards improving care transitions for PLWD and their care partners in the Laval CISSS and other regions of the province. This project will positively impact these individuals’ follow-up, support, and quality of life. Among other things, the training of professionals on dementia, the early referral to community resources, and the structured disclosure of dementia diagnosis are strategies that will improve care and service trajectories (i.e., receiving the right services, at the right time, in the right place and with the right professionals) [48].

Strengths and limitations should be highlighted. First, our living lab approach differs from traditional research on aging by facilitating co-design and co-evaluation of innovations, with the involvement of key partners. Our research team is multidisciplinary and composed of highly qualified experts from various fields, including dementia, healthcare services organizations and implementation and evaluation science. Moreover, the Laval-ROSA Transilab has excellent potential for transferability, as we intend to support the replicability of our methods and innovations to other healthcare organizations. The project relies on a complex multi-method approach involving multiple rounds of data collection.

Regarding limitations, the project's scope is important, which may lead to participant pressure or prevent the respect of our timeline. Thus, we developed three main strategies to address these challenges: 1) a research coordinator will ensure coordination and communication within the project components and actors; 2) we will also spread our activities among the 19 FMGs in the Laval area instead of focusing on the same few FMGs; 3) we will combine our data collection process as much as possible within the evaluation activities and synchronize them with the already planned clinical and managerial activities of the Laval CISSS; 4) we will focus on remote data collection procedures, such as videoconference calls. In addition, the COVID-19 pandemic has particularly destabilized practices in healthcare organizations, and several consequences are still being felt. There is, therefore, unpredictability in the ability of the organization and the professionals to fully engage in these innovations, exposing us to implementation challenges that will be alleviated with proper planning and close work with our partners at every stage.

The Laval-ROSA Transilab will directly benefit PLWD, their care partners, and healthcare professionals through its structuring actions. The latter will be better prepared to conduct an often-complex follow-up and benefit from a facilitated communication structure. Ultimately, improved coordination of transitions between FMGs, home care, and community services from the time of the dementia diagnosis will allow for a reduction in emergency room visits, potentially avoidable hospitalizations, re-hospitalizations, and unwanted or necessary admissions to long-term care.

## Data Availability

The datasets generated during and/or analyzed during the present study will be available from the corresponding author on reasonable request.

## Abbreviations

CPCSSN: Canadian Primary Care Sentinel Surveillance Network
FMGs: Family medicine groups
CISSS: Integrated health and social services center
LHS: Learning health system
PLWD: People living with dementia
QAP: Quebec Alzheimer Plan
QICDSS: Quebec Integrated Chronic Disease Surveillance System
ROSA: Research on Organization of Healthcare Services for Alzheimers
